# Immune checkpoint inhibitors and the risk of major atherosclerotic cardiovascular events in patients with high-risk or advanced melanoma: a retrospective cohort study

**DOI:** 10.1186/s40959-022-00149-8

**Published:** 2022-12-02

**Authors:** Charlie Wang, Sophia Zoungas, Mabel Yan, Rory Wolfe, Andrew Haydon, Mark Shackleton, Mark Voskoboynik, Maggie Moore, Miles C. Andrews, Stephen J. Nicholls, Victoria Mar

**Affiliations:** 1 Victorian Melanoma Service, The Alfred Hospital, 55 Commercial Road, Melbourne, Victoria 3004 Australia; 2 School of Public Health and Preventive Medicine, Monash University, Melbourne, Victoria Australia; 3 Department of Medical Oncology, The Alfred Hospital, Melbourne, Victoria Australia; 4 Department of Medicine - Alfred, Monash University, Melbourne, Victoria Australia; 5 MonashHeart, Monash Health, Clayton, Victoria Australia; 6 Monash Cardiovascular Research Centre, Victorian Heart Institute, Monash University, Clayton, Victoria Australia

## Abstract

**Background:**

Immune checkpoint inhibitors (ICI) are associated with immune-mediated adverse effects, potentially involving any organ. ICI has also been associated with an increased risk of cardiovascular disease in cancer populations.

**Objective:**

To characterize the incidence and risk of major atherosclerotic cardiovascular events associated with ICI use in a high-risk and advanced melanoma population.

**Methods:**

We conducted a retrospective cohort study of patients with high-risk or advanced melanoma (AJCC stage II, III or IV) presenting to an academic tertiary hospital between 2015–2020. The main outcome was major atherosclerotic cardiovascular events (MACE) including acute myocardial infarction, ischemic stroke, acute limb ischemia and coronary revascularization.

**Results:**

The study cohort consisted of 646 patients, including 289 who had been treated with ICI. The incidence of MACE was higher in the ICI treated group (3.6 vs. 0.9 events per 100-person years). After adjusting for age, sex, smoking history and prior BRAF and/or MEK inhibitor use, ICI treatment was associated with an increased risk of MACE (HR_adj_ 2.8, 95% CI 1.1–6.9, *p* = 0.03). Elevated risk was especially pronounced in patients with a past history of MACE (HR 14.4, 95% CI 1.9–112.3, *p* = 0.01).

**Conclusion:**

Patients with high-risk or advanced melanoma are at an increased risk of atherosclerotic cardiovascular events following ICI treatment, particularly those with a history of cardiovascular disease.

**Supplementary Information:**

The online version contains supplementary material available at 10.1186/s40959-022-00149-8.

## Introduction

Malignant melanoma is a common and serious form of skin cancer, with more than 300,000 cases diagnosed worldwide in 2020. Global incidence is increasing and it is predicted to become the second commonest malignancy in the US by 2040 [1, 2]. In the last decade, advances in cancer immunotherapy have transformed the treatment of advanced and high-risk melanoma. In particular, immune checkpoint inhibitors (ICI) that target cytotoxic T lymphocyte antigen 4 (CTLA4), such as ipilimumab, and programmed death 1 (PD-1), such as nivolumab and pembrolizumab, are now standards of care in stage IIIB, IIIC, IIID and IV disease. Indications for ICI in melanoma may be expanding; pembrolizumab was approved by the FDA for resected stage IIB and IIC malignant melanoma in December 2021, on the basis of improved recurrence-free survival in the KEYNOTE-716 trial [3].

With improved survival and greater exposure to systemic treatments, melanoma survivorship issues are increasingly important, including management of late effects of treatment. Although generally well tolerated, ICI may be associated with immune-related adverse events such as colitis, arthritis, hepatitis, thyroiditis, dermatitis, and hypophysitis, due in large part to induction of self-reactive T-cells. Some of these complications can persist even after cessation of ICI, requiring long-term or repeated immunosuppression or pharmacological endocrine replacement therapy.

Augmented immune activity can also have unintended effects on the heart and vasculature. Immune-related myocarditis is the best-known cardiac toxicity of ICI therapy, and although uncommon, is associated with significant mortality [4]. Furthermore, an increased risk of atherosclerotic cardiovascular diseases has been postulated, as PD-1 and CTLA-4 checkpoints are also regulators of atherosclerotic inflammation [5–7]. Indeed, multi-system immune-mediated diseases, such as rheumatoid arthritis, psoriasis systemic lupus erythematosus and other vasculitides, are associated with an increased risk of cardiovascular disease and atherosclerosis [8]. From in vitro studies, T-cells are highly prevalent in atherosclerotic lesions, and patients with advanced plaques may exhibit a distinct subset of activated CD4 + T-cells [9].

Recently, ICI have been associated with an increased risk of atherosclerosis, including myocardial infarction, cerebrovascular disease and dyslipidemia in meta-analyses of controlled trials and some observational studies [5, 10, 11]. However, these studies examined heterogeneous cancer populations with variable exposures to other cardiotoxic chemotherapeutics and systemic agents. Nevertheless, from the population-based Surveillance, Epidemiology and End Results registry, melanoma survivors were at increased risk of long-term cardiovascular mortality, particularly those with regional and distant disease [12].

The aim of our study was to characterize the real-world incidence and risk of atherosclerotic cardiovascular events associated with ICI in a high-risk and advanced melanoma population (AJCC Stage II-IV), and to identify factors that may help risk-stratify patients.

## Methods

### Participants and study period

We conducted a retrospective cohort study of patients that were enrolled in the Victorian Melanoma Service (VMS) clinical database between 1^st^ January 2015 and 30^th^ September 2020. The VMS is a statewide referral service for malignant melanoma, and provides care to 20–25% of all patients with a new diagnosis of melanoma in Victoria, Australia. The VMS prospectively collects data on all patients; including melanoma characteristics, recurrence, death and use of treatments such as ICI, targeted therapy and radiotherapy.

### Inclusion, exclusion criteria and study period

We defined two groups of VMS patients for this study: ICI treated and non-ICI treated groups. For the non-ICI treated group, we included all patients that were enrolled in the VMS database with a stage II (after sentinel lymph node biopsy), III or IV cutaneous malignant melanoma and did not receive any ICI therapy prior to or during the study period. For the ICI treated group, we included all VMS patients that had commenced and received at least one dose or treatment cycle of ICI (e.g. CTLA-4, PD-1, PD-L1 inhibitors) during the study period. Patients in both groups may have been treated with targeted agents (i.e. BRAF and/or MEK inhibitor therapies). We excluded patients that were enrolled in a placebo-controlled trial (and treatment allocation to ICI was unknown), patients treated with ICI for another malignancy, patients with no clinical follow-up after baseline. Baseline for the study period was from ICI commencement (for the ICI treated group) or date of initial presentation to VMS (for the non-ICI treated group. The study period ended at the patient’s date of death or last clinical contact (up until 30^th^ November 2021).

### Exposure and covariates of interest

We undertook a detailed chart review for all patients in our study. For ICI exposure data, we extracted details of treatment including drug name, commencement date, and number of lines of therapy. Data on potential confounding variables were also collected, including patient demographics (age, sex, BMI), cardiovascular medications (aspirin and statins), and cardiovascular risk factors (diabetes, hypertension, hyperlipidaemia, smoking status, past history of major atherosclerotic cardiovascular events as defined below). Data on melanoma characteristics and staging were extracted from the VMS Clinical Database. We also contacted either patients by telephone or their primary care physicians to obtain further clinical information up until 30^th^ November 2021.

### Outcomes

The primary outcome was the occurrence of any major atherosclerotic cardiovascular event (MACE). In this study, MACE was defined as a composite of acute myocardial infarction, ischemic stroke, acute limb ischemia, and coronary or other arterial revascularization procedure. Potential events were identified from imaging or operation reports, hospital discharge summaries, or cause of death certificates. Two clinicians (CW and MY) independently confirmed each event based on standardized definitions of myocardial infarction, ischemic stroke and acute limb ischemia (see Supplementary Material) [13–15]. Any disagreements were resolved by discussion and consensus.

### Data collection, storage and ethics

Study data were collected and managed using REDCap electronic data capture tools hosted by The Alfred Hospital. “REDCap is a secure, web-based software platform designed to support data capture for research studies, providing 1) an intuitive interface for validated data capture; 2) audit trails for tracking data manipulation and export procedures; 3) automated export procedures for seamless data downloads to common statistical packages; and 4) procedures for data integration and interoperability with external sources” [16].

Approval for this study was obtained from The Alfred Hospital Human Ethics Committee (Project 183/21). This study was conducted in accordance with the ethics standards of the institutional research committee and the Declaration of Helsinki.

### Statistical analysis

All continuous variables followed skewed distributions and were summarised as median (with interquartile range). We performed univariate analysis to compare covariates and baseline characteristics between the ICI treated and non-ICI treated groups, including Chi-squared test for categorical variables and Mann Whitney U test for continuous variables.

To determine whether ICI was associated with an increased risk of MACE, we performed unadjusted and adjusted Cox proportional hazards regression analyses with ICI as the exposure variable and time to first occurrence of MACE as the outcome. The adjusted model included age, sex and any unbalanced baseline co-variates or risk factors (*p* < 0.2 on univariate analysis). We assessed the assumptions of the Cox models by inspecting Schoenfeld residuals and log–log plots of survival and where the proportional hazards assumption was violated, the Cox model’s baseline hazard was stratified by the variable that did not conform to proportional hazards.

We performed subgroup and stratified analyses by sex, age ≥ 75, past history of MACE, smoking history, disease extent at baseline (distant vs. locoregional disease), and any exposure to BRAF and/or MEK inhibitors. We tested the interaction between ICI and covariates to assess for evidence of effect modification (if *p* < 0.05 on tests of interaction). Statistical analyses were performed with Stata version 17 (StataCorp, College Station, Texas).

## Results

### Baseline demographic, cardiovascular and melanoma characteristics

From the VMS clinical database, 646 patients were eligible for this study—including 289 and 357 ICI treated and non-ICI treated patients, respectively. 36 patients were excluded; 24 patients were enrolled in a placebo-controlled ICI clinical trial and treatment allocation was unknown, 10 had no follow after baseline, and 2 received ICI treatment for another malignant indication. The median follow-up was similar between groups; 23.2 months and 23.4 months in the ICI treated and non-ICI treated groups, respectively.

Baseline demographics and cardiovascular risk factors are summarized in Table 1, and melanoma tumour staging is shown in Table 2. Overall, groups were similar in terms of median BMI, hypertension, dyslipidemia, diabetes, past history of MACE, aspirin and statin use. Tumour characteristics such as Breslow thickness, ulceration and histological subtype were also similar between groups. The ICI treated group had a higher proportion of male patients (72.0% vs. 62.5%, *p* = 0.01) and greater proportion of patients with advanced stage disease (See Table 2).

**Table 1:** Baseline demographic characteristics and cardiovascular risk factors

Baseline	ICI treated (*n* = 289)	Non-ICI treated (*n* = 357)	*p*-value
Median age (years), IQR	67.9 (57.4—77.3)	66.1 (55.1—75.7)	0.09^a^
Male (%)	72.0	62.5	0.01^b^
Median BMI, IQR	27.7 (24.4—31.9)	27.2 (24.6—30.9)	0.41^a^
Current smoker (%)	11.7	13.0	0.66^b^
Ever smoker (%)	48.1	44.3	0.08^b^
Hypertension (%)	45.4	41.9	0.38^b^
Dyslipidemia (%)	26.3	26.4	0.98^b^
Diabetes (%)	12.1	12.4	0.92^b^
Past History of MACE (%)	9.8	13.5	0.15^b^
Aspirin use^c^ (%)	17.4	15.6	0.53^b^
Statin use^c^ (%)	30.0	27.5	0.49^b^
Median follow-up (months), IQR	23.2 (10.2 – 37.6)	23.4 (11.3 – 40.1)	0.69^a^

^a^Mann–Whitney U test

^b^Chi-squared test

^c^Defined as documented aspirin or statin use at study baseline

*MACE* Major atherosclerotic cardiovascular event

**Table 2:** Melanoma characteristics at baseline

	**ICI treated (*n* = 289)**	**Non-ICI treated (*n* = 357)**	***p*-value**
Melanoma characteristics			
Breslow in mm (IQR)	2.7 (1.5—4.7)	2.8 (2—4.2)	0.829^a^
Ulceration (%)	43.0% (108)	38.4% (129)	0.257^b^
Histologic subtype			
SSM (%)	43.0	41.0	
Nodular (%)	29.6	34.0	
LMM (%)	8.1	4.9	
ALM (%)	4.5	5.3	
Other (%)	14.8	14.8	
AJCC 8^th^ Edition Stage at baseline			
IIA	0	137	
IIB	1	56	
IIC	3	31	
IIIA	2	21	
IIIB	52	55	
IIIC or IIID	94	48	
IV	137	9	

^a^Mann–Whitney U test

^b^Chi-squared test

*SSM* Superficial spreading melanoma, *LMM *Lentigo maligna melanoma, *ALM*Acral lentiginous melanoma, *AJCC* American Joint Committee on Cancer

In the ICI treated group, 63.0% and 41.5% of patients had received nivolumab and pembrolizumab, respectively. 74 (25.6%) patients had been treated with combination ipilimumab-nivolumab. There were four patients who had received ICI during a clinical trial (either nivolumab or ipilimumab), however treatment allocation was unknown. 63.7% of patients treated with ICI experienced an immune-related adverse event (IRAE). Data about IRAEs and concomitant immunomodulatory treatment is shown in Table 3.

**Table 3:** Melanoma systemic treatments and immune-related adverse events (IRAE)

	**ICI treated (*n* = 289)**	**Non-ICI treated (*n* = 357)**	***p*-value**
Pembrolizumab	41.5% (120)		
Nivolumab	63.0% (182)		
Combination Ipilimumab/Nivolumab	25.6% (74)		
Ipilimumab or Nivolumab (blinded)	1.4% (4)		
Other	1.0% (3)		
Any IRAE	63.7% (184)		
Cutaneous	26.0% (75)		
Thyroiditis	11.4% (33)		
Hypophysitis/Hypopituitarism	5.5% (16)		
Adrenal insufficiency/adrenalitis	3.1% (9)		
Enterocolitis	12.5% (36)		
Hepatitis	9.7% (28)		
Pneumonitis	3.5% (10)		
Arthritis	12.8% (37)		
Myocarditis	0.7% (2)		
Any corticosteroid use after ICI	46.4% (132)		
Chronic corticosteroid use (> 30 days) after ICI	37.8% (107)		
TNF-alpha inhibitor use after ICI	5.5% (16)		
Any BRAF ± MEK inhibitor use	12.5% (36)	10% (36)	0.33
BRAF ± MEK inhibitor use before baseline	8.0% (23)	0.0% (0)	< 0.01
BRAF ± MEK inhibitor use after baseline	4.5% (13)	12.5% (36)	< 0.01

### Major atherosclerotic cardiovascular events in the ICI group

In total, there were 29 events in 23 patients; including 22 events in 16 ICI treated patients. In the ICI treated group, acute myocardial infarction occurred in 8 patients (12 events), ischemic stroke in 5 patients (7 events), acute limb ischemia in 2 patients (3 events) and percutaneous coronary intervention was required in one patient with unstable angina. 50% of ICI treated patients experienced MACE within 6 months of ICI commencement, and the median age was 78.7 years when MACE occurred.

Among the ICI treated patients who experienced MACE, 9 patients had been treated with Pembrolizumab, 3 patients had been treated with nivolumab (monotherapy), 2 patients had been treated with combination ipilimumab/nivolumab, and 1 patient had been treated with either ipilimumab or nivolumab (blinded). Only one patient had received BRAF and/or MEK inhibitor therapy prior to MACE. Furthermore, 9 patients experienced IRAEs, and 7 received corticosteroid therapy prior to MACE. None were treated with methotrexate or a biological agent prior to MACE.

In the non-ICI treated, 4 patients required coronary revascularization for unstable angina, 2 patients experienced acute myocardial infarction, and 1 experienced acute limb ischemia.

### Risk of major atherosclerotic cardiovascular events

The incidence of MACE in the ICI treated group was 3.6 events per 100-person years, compared to 0.9 events per 100 person-years in the non-ICI treated group (*p* < 0.001). In an unadjusted analysis, the risk of MACE was increased in the ICI treated group (HR 3.1, 95% CI 1.3 – 7.2, *p* = 0.01). This increased risk persisted (HR_adj_ 2.8, 95% CI 1.1 – 6.9, *p* = 0.03) after fitting a stratified Cox model (sex did not satisfy the proportional hazard assumption), and adjusting for age, history of tobacco smoking (ever or never), and prior BRAF and/or MEK inhibitor use.

On subgroup analyses (See Table 4), an increased risk of MACE was observed when restricted to patients with locoregional disease (Stage II or III) at baseline (HR 3.0, 95% CI 1.1 – 7.9, *p* = 0.03), male patients (HR 4.2, 95% CI 1.4 – 12.7, *p* = 0.01), patients without BRAF and/or MEK inhibitor exposure (HR 3.5 95% CI 1.3 – 8.9, *p* = 0.01) and patients who have ever smoked (HR 3.5 95% CI 1.0 – 12.5, *p* = 0.05). None of these subgroups had statistically significant interactions.

**Table 4:** Time-to-first MACE event—Subgroup analysis

	**Subgroups**	**No. of patients**	**Unadjusted HR (95% CI)**	***p*-value**	***p*-value for interaction**
Past History of MACE	Yes	74	14.4 (1.9—112.3)	0.01	0.03
	No	571	1.14 (0.4—3.8)	0.82	
Sex	Male	431	4.2 (1.4—12.7)	0.01	0.13
	Female	215	0.8 (0.1—8.1)	0.86	
Age	≥ 75 years	192	5.21 (1.2—22.9)	0.03	0.23
	< 75 years	454	1.77 (0.6—13.3)	0.33	
Ever smoker (current or former)	Yes	268	3.5 (1.0—12.5)	0.05	0.73
	No	295	2.58 (0.6—10.6)	0.19	
Disease extent at baseline	Locoregional disease (Stage II/III)	500	2.96 (1.1—7.9)	0.03	NA
	Distant metastatic disease (Stage IV)	146	NA	NA	
BRAF ± MEK inhibitor use	Yes	72	1.2 (0.1—19.0)	0.9	0.47
	No	573	3.46 (1.3—8.9)	0.01	

*HR* Hazard Ratio, *CI* Confidence interval

Among patients with a past history of MACE, ICI treatment was associated with a significantly elevated risk (HR 14.4, 95% CI 1.9 – 112.3, *p* = 0.01). Conversely, there was no increased risk in patients without a past history of MACE (HR 1.1, 95% CI 0.4 – 3.8, *p* = 0.82). As such, past history of MACE was a significant effect modifier (*p*-value for interactio*n* = 0.03).

## Discussion

Our study of patients with high-risk and advanced melanoma confirms an association between ICI and an increased risk of MACE (myocardial infarction/acute coronary syndrome, ischemic stroke and acute limb ischemia). Our findings are consistent with a large retrospective, single-centre cohort study and systematic review and meta-analyses examining ICI use in all malignancies [5, 11]. In a study of 5684 subjects with various malignancies, Drobni et al. reported that 4.2% of patients experienced a major cardiovascular event (defined as ischemic stroke, myocardial infarction and coronary revascularization), which represented a three-fold increased risk compared to matched controls [5]. Furthermore, computerized tomographic coronary angiography in 40 melanoma patients from this cohort demonstrated a three-fold increase in total plaque progression (6.7% vs. 2.1%) in ICI treated patients. Meanwhile, a safety meta-analysis of randomized controlled trials reported a modest association with myocardial infarction (OR 1.56, 95% CI 1.01 – 2.26) and cerebral ischemia (OR 1.51, 95% CI 1.01 – 2.26).

The mechanism for this increased risk is thought to involve PD-1/PD-L1 pathways, which are involved in suppressing anti-tumour immunity as well as regulating atherogenic T-cell activity. In vivo studies show that PD-L1/2 deficient mice had significantly increased aortic plaque burden as well as increased numbers of CD4 + and CD8 + T-cells within atherosclerotic lesions and increased serum concentrations of interferon-gamma [17]. Furthermore, administration of anti-PD-1 antibodies in these mice further increased lesional inflammation and T-cell recruitment [6]. The converse is also true; stimulation of PD-1 signalling in mice inhibits atherosclerosis by modulating T-cell activity – reducing interferon-gamma producing CD4 + T cells and increasing atheroprotective IL-10 secreting CD4 + T-cells. Similarly, CTLA-4 inhibition has also been demonstrated to promote atherosclerotic plaque progression and aggravation in mice [7]. A notable finding in our study is that half of the MACE occurred within six months of ICI commencement, which has also been suggested in other case series and observational studies [18–21]. This suggests that a short-term increase in cardiovascular risk may be attributable to plaque destabilization and/or altered vasoreactivity.

Notably, the incidence of MACE was high amongst ICI treated patient in our study (3.6 event per 100 person years), but is consistent with other observational studies [5, 20, 21]. The rate of MACE in the non-ICI treated group was 0.9 events per 100 person-years, which is comparable to the rate of major adverse cardiac events (defined as non-fatal myocardial infarction, ischemic stroke, hospitalization for heart failure and cardiovascular death) in healthy elderly from the ASPREE trial [22]. Nevertheless, the rate of MACE in ICI treated patients may still be underestimated in our study. Recently, a prospective cohort study found that 10% of patients treated with ICI had Type 2 myocardial infarction secondary to other causes within 10 cycles of immunotherapy, when monitored 2–4 weekly with high sensitivity troponin testing for the detection of immune-related myocarditis [23].

In our study, ICI treated patients with a past history of MACE were at greatest risk. This may be an important consideration in relation to risk stratification and assessment of suitability for ICI therapy. Ultimately, a history of cardiovascular disease should not preclude the use of ICI in patients with advanced stage melanoma, as prognosis is poor if untreated. However, caution may be necessary in the adjuvant setting following curative resection of melanoma (Stage II and III). Furthermore, risk factor modification and/or cardiovascular screening (stress-testing, cardiac angiography) prior to ICI commencement may be advisable, particularly for those with a past history of MACE and where clinical circumstances permit such testing to be performed prior to initiation of therapy. Indeed, the NHMRC-funded SOCRATES randomized controlled trial is underway, which will evaluate the effects of statin on changes in coronary atherosclerosis in melanoma patients treated with immune checkpoint inhibitors [24].

Notably, Pembrolizumab was the commonest ICI treatment prior to MACE. PD-1 inhibitors (nivolumab and pembrolizumab) are essentially identical with regards to mechanism of action, IgG subclass, binding specificity and affinity [25]. Some differences may arise in the epitope-binding variable regions of the antibody; although this is unlikely to account for significant drug-dependent differences. From clinical studies, no significant differences in efficacy or toxicities between pembrolizumab and nivolumab are observed in melanoma [26, 27]. Nonetheless, exploration of possible differences in cardiovascular toxicity profile between immune checkpoint inhibitors (CTLA4, PD-1, PDL-1) may be warranted.

Our study has a number of limitations. Firstly, the total number of events in our study is low. Although, our results were significant, it is possible that our risk estimate is imprecise. Nevertheless, a previous large retrospective cohort study has also reported a threefold increased risk in ICI treated patients with various malignancies. Secondly, our study may be liable to bias due to differences in melanoma staging between groups. In the ICI treated group, almost half of the patients had Stage IV (distant metastatic disease) at baseline compared to 2.3% in the non-ICI treated group. This difference in staging between groups is difficult to control for in real world studies, as nowadays, the majority of patients with Stages III and IV malignant melanoma will receive ICI. Nonetheless, we performed subgroup analysis on patients with only locoregional disease (Stage II/III) at baseline, and effect sizes were similar. Finally, our study is limited by its retrospective design; in particular, medication dosages and measures of control of cardiovascular risk factors such as blood pressure readings, glycated hemoglobin levels were unavailable or inconsistently measured. Strengths of our study include analysis of melanoma patients in a real-world setting and the use of standardized definitions in outcome assessment.

## Conclusion

Our real world study confirms an association between major atherosclerotic cardiovascular events and ICI treatment in a population of high-risk and advanced melanoma patients. Events typically occurred within six months of ICI commencement indicating increased short-term risk, possibly related to plaque destabilization. The long-term risk of cardiovascular disease and mortality associated with ICI treatment is unclear and warrants prospective evaluation. Furthermore, our data suggests patients with a significant history of cardiovascular disease are at greatest risk. As such, melanoma clinicians should consider risk factor modification and/or cardiovascular screening prior to ICI commencement in these individuals.

## Supplementary Information

**Additional file 1:** **Supplementary Material.** Definitions used for outcome assessment.

## Data Availability

Dataset used for this study will be made available upon reasonable request to the corresponding author.
