# Supplementary material for: Immune checkpoint inhibitors and the risk of major atherosclerotic cardiovascular events in patients with high-risk or advanced melanoma: a retrospective cohort study
Source: Cardiooncology. 2022 Dec 2;8:23. doi: 10.1186/s40959-022-00149-8 (PMC9716700; doi:10.1186/s40959-022-00149-8)
Supplement: Supplementary file 1 — Additional file 1: Supplementary Material. Definitions used for outcome assessment. [file 40959_2022_149_MOESM1_ESM.docx]

**Supplementary Material: Definitions used for outcome assessment**

**Acute myocardial infarction**

Acute myocardial infarction (MI) was defined under the following conditions

- 1) Detection of a rise and/or fall of cardiac biomarker values [preferably cardiac troponin (cTn)] with at least one value above the 99th percentile upper reference limit and with at least one of the following:
  - Symptoms of ischemia
  - New or presumed new significant ST-segment–T wave (ST–T) changes or new left bundle branch block (LBBB)
  - Development of pathological Q waves in the ECG.
  - Imaging evidence of new loss of viable myocardium or new regional wall motion abnormality
  - Identification of an intracoronary thrombus by angiography or autopsy
- 2) Cardiac death with symptoms suggestive of myocardial ischemia and presumed new ischemic ECG changes or new LBBB, but death occurred before cardiac biomarkers were obtained, or before cardiac biomarker values would be increased
- 3) Percutaneous coronary intervention (PCI) related MI: Defined by elevation of cardiac troponin values (5 x >99th percentile) in patients with normal baseline values (≤99th percentile) or a rise of troponin values >20% if the baseline values are elevated and are stable or falling. In addition, either (i) symptoms suggestive of myocardial ischemia or (ii) new ischemic ECG changes or (iii) angiographic findings consistent with a procedural complication or (iv) imaging demonstration of new loss of viable myocardium or new regional wall motion abnormality are required or (v) angiographic or autopsy demonstration of stent thrombosis
- 4) Coronary artery bypass grafting related MI: Defined by elevation of cardiac biomarker values (>10 × 99th percentile) in patients with normal baseline troponin values (≤99th percentile). In addition, either (i) new pathological Q waves or new LBBB, or (ii) angiographic documented new graft or new native coronary artery occlusion, or (iii) imaging evidence of new loss of viable myocardium or new regional wall motion abnormality.

*Adapted from Third Universal Definition of Myocardial Infarction (Thygesen et al. Circulation 2012)*

**Ischemic Stroke**

An episode of neurological dysfunction caused by focal cerebral, spinal or retinal infarction, as evidenced by:
1) Pathological, imaging or other objective of cerebral, spinal cord or retinal focal ischemic injury in a defined vascular distribution
2) Clinical evidence of clinical evidence of cerebral, spinal cord, or retinal focal ischemic injury based on symptoms persisting for 24 hours or more or until death, and other etiologies excluded.

*Adapted from An updated definition of stroke for the 21^st^ century: a statement for healthcare professionals from the American Heart Association/American Stroke Association (Sacco et al. Stroke 2013)*

**Acute limb ischemia**

Acute limb ischemia was defined as a clinical history of sudden significant worsening of limb perfusion requiring hospitalization and:

- A new pulse deficit with associated rest pain, pallor, paraesthesia, or paralysis and confirmation of arterial obstruction either by imaging, limb hemodynamics, intraoperative or pathological evaluation

OR

- Requiring thrombolysis, thrombectomy or urgent revascularization

*Adapted from VOYAGER PAD Trial (Bonaca et al. New England Journal of Medicine 2020)*
